# The Effect of Input DNA Copy Number on Genotype Call and Characterising SNP Markers in the Humpback Whale Genome Using a Nanofluidic Array

**DOI:** 10.1371/journal.pone.0039181

**Published:** 2012-06-20

**Authors:** Somanath Bhat, Andrea M. Polanowski, Mike C. Double, Simon N. Jarman, Kerry R. Emslie

**Affiliations:** 1 National Measurement Institute, Lindfield, New South Wales, Australia; 2 Autralian Marine Mammal Centre, Australian Antarctic Division, Kingston, Tasmania, Australia; Auburn University, United States of America

## Abstract

Recent advances in nanofluidic technologies have enabled the use of Integrated Fluidic Circuits (IFCs) for high-throughput Single Nucleotide Polymorphism (SNP) genotyping (GT). In this study, we implemented and validated a relatively low cost nanofluidic system for SNP-GT with and without Specific Target Amplification (STA). As proof of principle, we first validated the effect of input DNA copy number on genotype call rate using well characterised, digital PCR (dPCR) quantified human genomic DNA samples and then implemented the validated method to genotype 45 SNPs in the humpback whale, *Megaptera novaeangliae,* nuclear genome. When STA was not incorporated, for a homozygous human DNA sample, reaction chambers containing, on average 9 to 97 copies, showed 100% call rate and accuracy. Below 9 copies, the call rate decreased, and at one copy it was 40%. For a heterozygous human DNA sample, the call rate decreased from 100% to 21% when predicted copies per reaction chamber decreased from 38 copies to one copy. The tightness of genotype clusters on a scatter plot also decreased. In contrast, when the same samples were subjected to STA prior to genotyping a call rate and a call accuracy of 100% were achieved. Our results demonstrate that low input DNA copy number affects the quality of data generated, in particular for a heterozygous sample. Similar to human genomic DNA, a call rate and a call accuracy of 100% was achieved with whale genomic DNA samples following multiplex STA using either 15 or 45 SNP-GT assays. These calls were 100% concordant with their true genotypes determined by an independent method, suggesting that the nanofluidic system is a reliable platform for executing call rates with high accuracy and concordance in genomic sequences derived from biological tissue.

## Introduction

Single Nucleotide Polymorphism, or SNP, is the most common form of variation which occurs when a single nucleotide (A, T, G or C) in the genome is changed[1]. SNP-genotyping (SNP-GT) is rapidly growing as a useful tool in many scientific disciplines including personalised medicine[2], forensics[3], plant and animal biotechnology[4], [5]. Genome-wide association studies utilizing SNPs as markers have enabled identification of genes that underline complex disorders[6]. Depending on the location, a SNP might have consequences at the phenotypic level. Most SNPs are located in the non-coding regions of the genome and have no direct known impact on the phenotype of an individual[7] or cell function[1]. However, some SNPs regardless of their location may pre-dispose the individual to a certain disease or influence their response to drug[3], [8]. To identify an association between a SNP and a particular disease or genetic trait, researchers need high throughput, cost effective and accurate approaches to screen vast numbers of samples for numerous SNPs. One such approach is the Fluidigm Dynamic Array^TM^ platform (nanofluidic based genotyping system) which can handle medium throughput multiplexing[9].

The 48.48GT Dynamic Array^TM^ technology (Fluidigm, South San Francisco) allows simultaneous analysis of 48 different SNPs in 48 individual samples using TaqMan^®^ SNP-GT assays. The key to the efficiency of this approach is the chip architecture. The chip consists of a matrix of channels, chambers, and integrated valves finely patterned into layers of silicone in the nanofluidic chip. The valves partition sample / assay combinations into a total of 2,304 (48×48) individual reaction chambers prior to thermal cycling.

The genotype for a particular SNP is either homozygous (pp or qq) or heterozygous (pq) and the genotype designated to a SNP following analysis is referred to as the genotype call. The genotype call quality can vary when the amount and/or the quality of the input DNA is not ideal and this may lead to either an incorrect genotype call or No Call for a particular SNP. Specific Target Amplification (STA) can be used prior to genotyping to increase the input DNA copy number. In previous studies STA was performed for 14 cycles in a multiplex format[9], [10], [11]. Multiplex STA provides simultaneous amplification of many targets of interest in one reaction, thus increasing the assay throughput and allowing more efficient use of each DNA sample[5], [11], [12]. Multiplex reactions, however, need to be validated to ensure that all reactions are amplified efficiently.

In this study, we validated a relatively low cost nanofluidic system for SNP-GT. As proof of principle, we first evaluated the effect of input DNA copy number on genotype call rate using well characterised digital Polymerase Chain Reaction (dPCR)-quantified human genomic DNA samples with three different genotypes for a single SNP. Accurate quantification of DNA samples using dPCR combined with sample gravimetric dilutions prior to mixing with other reagents, enabled dispensing of a predicted number of DNA copies into each reaction chamber. The sourced human genomic DNA samples and the SNP assay have been previously used by Wang et al[9]. For samples with low starting input DNA concentration, we validated both simplex and multiplex STA prior to genotyping. This validation approach was then applied to twelve DNA samples that had been extracted from epidermis biopsies of humpback whales, *Megaptera novaeangliae*. STA initially used fifteen SNP-GT assays previously validated by Polanowski *et* *al.* [13] and was subsequently adapted for 45 SNP-GT assays. Simplex STA was designed to test the specificity of each SNP assay. The genotype of each sample determined using the nanofluidic platform at the National Measurement Institute (NMI), was compared for concordance with the true genotypes determined by an independent method at the Australian Antarctic Division (AAD).

## Results and Discussion

### Evaluating Genotype Call Accuracy on Human Genomic DNA

The stock concentrations (copies/µL –haploid genome equivalents) of three human genomic DNA samples were determined by dPCR (n  =  3 or 4) to be 4.7×10^4^ copies/µL with a range of 0.3×10^4^ (NA17313–‘qq’), 9.3×10^4^ copies/µL with a range of 0.25×10^4^ (NA17316–‘pq’) and 1.4×10^5^ copies/µL with a range of 0.046×10^5^ (NA17317–‘pp’), respectively. Note: Range is the difference between the largest and smallest values in a set of data and was used rather than standard deviation.

**Figure 1:**
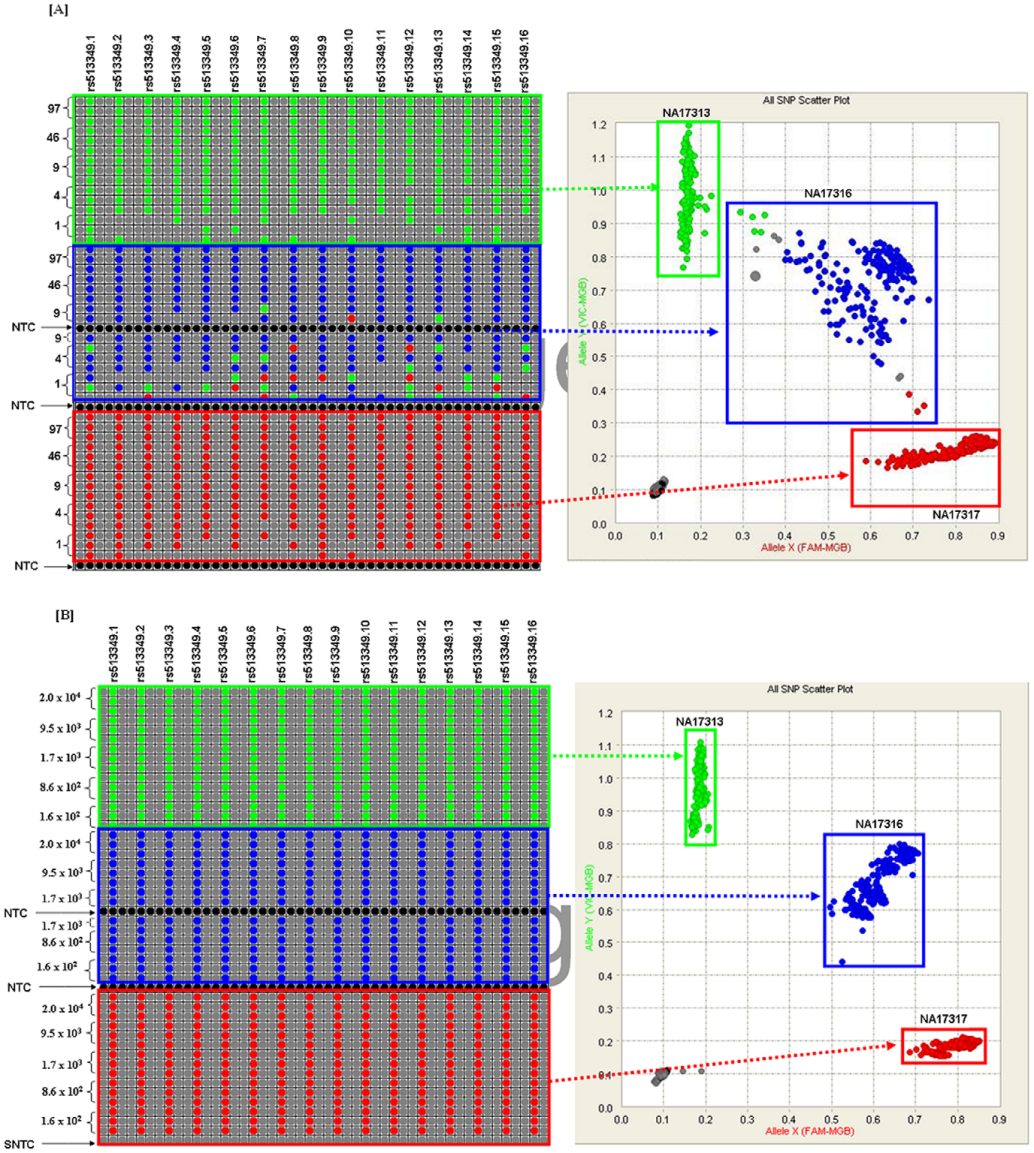
Effect of starting copy number on genotype call rate with and without STA. [A] and **[B]** Call map view and scatter plots of three human genomic DNA samples showing clustering (**pp**-‘**red**’, **qq**-‘**green**’ and **pq** ‘**blue**’) for a single SNP with and without STA. Black and Grey colors correspond to No Calls. The reaction chambers contained different copies ranging, on average, from approximately 97 to 1 copies(y) without STA and 2.0×10^4^ to 1.6×10^2^ copies with STA. SNP-GT assay (rs513349) was loaded into sixteen separate assay inlets evenly spaced across the 48.48GT array. The remaining inlets were loaded with a NPC as stated in the Methods section.

Preliminary analysis was performed to evaluate the effect of reaction copy number on call rate accuracy prior to TaqMan^®^ SNP-GT. The dPCR-quantified samples with three different genotypes (pp, pq, qq) for a single SNP (rs513349) were gravimetrically diluted so that the estimated final copies per reaction chamber ranged between 1 and 97 copies (haploid genome equivalents). The final copies per reaction chamber were derived using equations 1, 5–6 (Table 1). A genotype call rate and call accuracy of 100% was observed for reactions that were estimated to contain ≥ 9 homozygous (pp or qq) copies of the genotype or ≥ 46 heterozygous (pq) copies of the genotype. Reactions containing less than 9 homozygous copies of the genotype had a No Call rate of 6 to 60% for NA17313 (qq) and 2 to 35% for NA17317 (pp). However, when a genotype was assigned to a chamber containing homozygous DNA, the assigned genotype was correct regardless of the estimated copy number. For the heterozygous sample (NA17316-pq), reaction chambers containing ≤ 9 copies had an incorrect call rate of 7 to 75% and a No Call rate of 4 to 33%. Clustering of genotype data points was tight for a homozygous call but was more widespread for a heterozygous call (Figure 1A).

**Table 1:** Derivation of DNA copy number concentration in the final reaction chamber with and without STA.

Factor	Units	Symbol	Equation number	Simplex STA	Multiplex STA	Without STA
Stock concentration of haploid genome	copies/µL	*C_H_*	1†	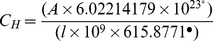		
STA reaction mixture pre-PCR	copies/µL	*E_S_*	2	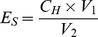		NA
STA reaction mixture post PCR	copies/µL	*E_SX_*	3	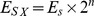		NA
Pooled STA reaction mixture post-PCR	copies/µL	*E_SP_*	4	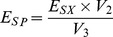	NA	NA
Diluted Multiplex STA reaction mixture	copies/µL	*E_GD_*	4^♦^	NA	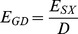	NA
GT sample mixture	copies/µL	*E_SM_*	5†	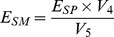	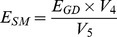	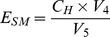
Reaction chamber (9∶1 mixture of sample and assay)	copies/reaction chamber	*E_RC_*	6†	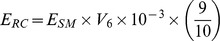		

Where *A* =  stock DNA concentration (ng/µL); *l* =  length in bp (human and whale genomes ∼3×10^9^ bp); V_1_ =  DNA sample volume for STA (1.25 µL); V_2_ =  STA mixture volume (5 µL); n =  Total number of PCR cycles; V_3_ =  Pooled mixture volume, which is derived by multiplying V_2_ and the number of SNP-GT assays (V_3_ = V_2_×SNP-GT assays); D =  Dilution factor (5- or 20- fold); V_4_ =  DNA sample volume for genotyping (2.1 µL); V_5_ =  GT sample solution volume (5 µL) and V_6_ =  Reaction chamber volume (6.75 nL)[9].

*E_SX_* was calculated assuming 100% PCR efficiency.

† Use equations 1, 5–6 when estimating copies/reaction chamber without STA.

Use equations 1–6 when estimating copies/reaction chamber with STA in simplex.

Use equations 1–3, 4^♦^ and 5–6 when estimating copies/reaction chamber with STA in Multiplex.

* The Avogadro number (6.02214179×10^23^) was taken from Mohr et al[20] (CODATA-2006).

•Average molecular weight of DNA base pair used was 615.8771[21].

NA – refers to Not Applicable.

In contrast, a call rate and a call accuracy of 100% were achieved when the same gravimetrically diluted samples were subjected to STA and then diluted 20- fold prior to genotyping resulting in 2.0×10^4^ to 1.6×10^2^ copies per reaction chamber. Clustering of the genotype calls for all three human genomic DNA samples was much tighter following STA than in the absence of STA (Figure 1B and 1A), suggesting that target enrichment improves the quality of results.

**Figure 2:**
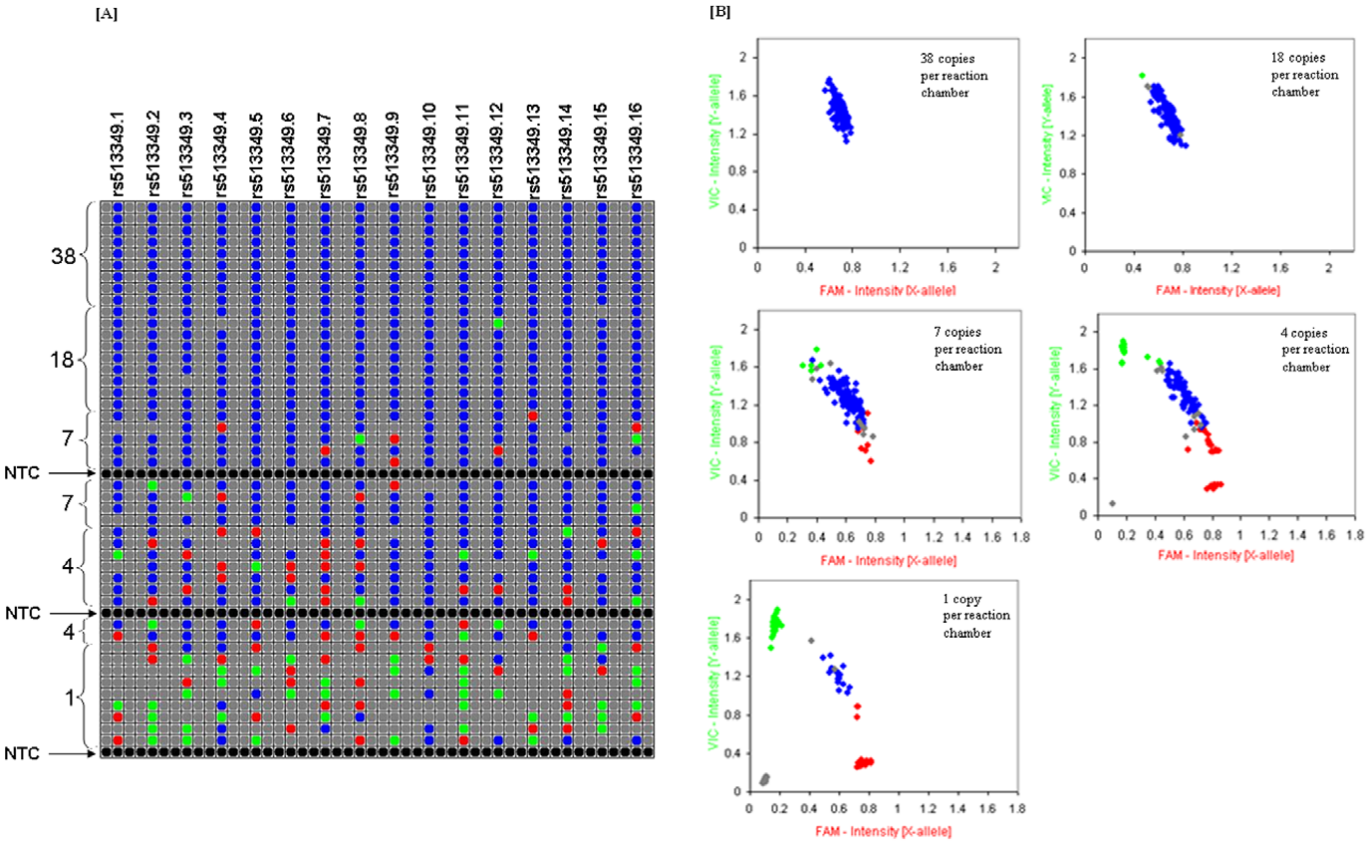
Effect of reaction DNA copy number on genotype call accuracy for a heterozygous (pq) sample. Call map view **[A]** and scatter plots **[B]** of the genotype calls from reaction chambers containing predicted 38, 18, 7, 4 and 1 copies(y). The genotype call (**pp**, **qq** and **pq**) for each reaction is denoted in ‘**red**’, ‘**green**’ and ‘**blue**’, respectively. No Call and NPC are denoted in ‘grey’ and NTCs in ‘black’. SNP-GT assay (rs513349) was loaded into sixteen separate assay inlets evenly spaced across the 48.48GT array. The remaining inlets were loaded with a NPC as stated in the Methods section.

Based on the preliminary findings, the effect of copy number on the heterozygous (pq) call accuracy was further investigated in more detail, by lowering the copies per reaction chamber and by increasing the total number of replicates for each dilution. Sample NA17316 (pq) was gravimetrically diluted resulting in 1 to 38 copies per reaction chamber. A call rate and a call accuracy of 100% were achieved when 38 copies per reaction chamber corresponding to ∼45 ng/µL of human genomic DNA solution used in sample preparation (∼90 ng in 2.1 µL of DNA in GT- sample solution) were present. At 18 copies per reaction chamber, which equates to 22 ng/µL, a call rate of 98% with a call accuracy of 99% was achieved (Table 2). As the number of copies per reaction chamber decreased below 18, the number of incorrect calls (pp, qq) and No Calls increased (Figure 2A) and the tightness of genotype clusters on the scatter plot decreased (Figure 2B). The call accuracy dropped from 88% at 7 copies to 33% at 1 copy (Table 2). The number of No Calls increased from 2 to 38% when the copies per reaction chamber decreased from 18 to 1 copy, suggesting low input DNA copy number affects the quality of data generated, in particular for a heterozygous (pq) sample. With a heterozygous locus, where two alleles are present, unequal sampling of the alleles at very low reaction copy numbers can result in failure to detect one (allele-drop out) or both of the alleles (locus drop-out)[14] leading to an incorrect or No Call. This would explain the observed increase in the number of incorrect and No Calls assigned for the heterozygous sample. Therefore, in order to minimise call error rate, it is important to maintain the allelic balance. Loss of heterozygosity (LOH), which is a common form of allelic imbalance, has been used to identify genomic regions that harbor tumor suppressor genes and to characterize tumor stages and progression[15]. To avoid misinterpretation of such data, it is critical that sufficient copies of the heterozygous locus are present in the genotyping assay.

**Table 2:** Effect of DNA copy number on reliability of genotype call data for a heterozygous human genomic DNA sample, NA17316.

	Copies per reaction chamber				
	1	4	7	18	38
**% Call rate – [All calls]** (1)	62	82	84	98	100
**% Call rate [pq calls]** (2)	21	60	74	97	100
**% Call accuracy [pq calls]** (3)	33	73	88	99	100

*Call rate and call accuracy (%) at different number of copies per reaction chamber and for pq calls were determined from 144 data points.*

^(1)^% Call rate [All Calls]  = 100*[(Total number of calls) / (Total number of calls + No Calls)].

^(2)^% Call rate [pq Calls]  =  100*[(Correct Calls) / (Total number of calls + No Calls)].

^(3)^% Call accuracy [pq Calls]  = 100*[(Correct Calls) / (Total number of calls)].

Previously Wang et al[9] showed that 50 copies per reaction chamber corresponding to ∼60 ng/µL of human genomic DNA is required to obtain a call rate of >99%. Further Chan et al[16], highlighted the importance of using purified samples to achieve ≥98% call rate. They observed low call rates (47.5% to 77.5%) when using unpurified clinical samples. By quantifying sample NA17316 using dPCR and performing gravimetric dilutions prior to genotyping analysis, an accurate starting copy number concentration of the sample was obtained. This enabled us to predict the number of copies of DNA required in each reaction chamber to obtain a call rate of 98–100%.

### Validation of Simplex and Multiplex STA Conditions Prior to Genotyping Whale Samples

**Figure 3:**
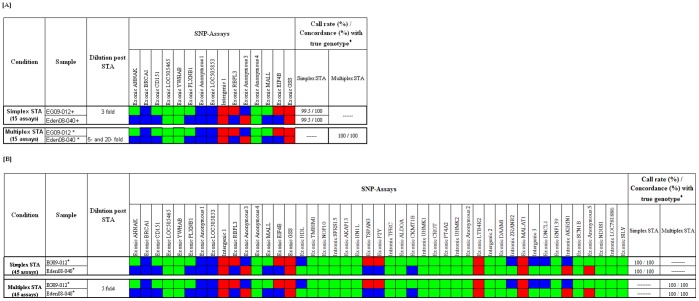
Summary genotype calls obtained for representative whale DNA samples. Genotype calls obtained for representative whale DNA samples extracted using CTAB or Maxwell^®^ tissue extraction kit and following simplex or multiplex STA using either 15 [A] or 45 SNP-GT assays [B]. The genotype call (**pp**, **qq** and **pq**) for each reaction are denoted in ‘**red**’, ‘**green**’ and ‘**blue**’, respectively. ‘+’ refers to samples extracted using CTAB method; '*' refers to each sample extracted using both CTAB and Maxwell^®^ tissue extraction kit; ‘♦’ refers to concordance with true genotype determined at AAD using an independent method. Note: Regardless of the approach used, genotypes for representative samples using either 15 or 45 SNP-GT assays were the same, as indicated by the same color. Samples EG09-004, EVH09-53, WA07-006 and WA07-003, extracted using CTAB or Maxwell^®^ tissue extraction kit were genotyped using 15 SNP-GT assays with and without STA and showed a 100% call rate and concordance (data not shown).

Twelve whale DNA samples were used for the validation process. The estimated DNA concentration of the samples varied significantly depending on the extraction method (Table 3). It was, therefore, necessary to incorporate an STA step prior to genotyping. The STA step was evaluated using either 15 or 45 SNP-GT assays in both simplex and multiplex format. Under simplex STA with 15 SNP-GT assays, for each sample an average call rate of greater than 99% was achieved on all four dilutions. Each dilution corresponded to different final copies per reaction chamber ranging from 17 to 662 (EG09-004), 58 to 2270 (EG09-012), and 49 to 1900 (Eden08-040), respectively. Similarly, using 45 SNP-GT assays, a call rate of 100% was achieved for both samples, EG09-012 (227 copies per reaction chamber) and Eden08-040 (189 copies per reaction chamber) (Figure 3A and 3B). The genotype calls showed 100% match with the Illumina transcriptome sequence data obtained at AAD[13].

**Table 3:** Samples analysed using the SNP-GT nanofluidic system.

Sample	Concentration (ng/µL)	
	Maxwell^®^ tissue DNA extraction Kit	CTAB
EG09-004	6	17
EG09-012	3	60
EVH09-53	10	7
Eden08-040	9	50
WA 07-006	4	24
WA 07-033	1	8

For multiplex STA, a call rate and a call accuracy of 100% was achieved for all samples when 15 SNP-GT assays were used (Figure 3A). Similarly, using 45 SNP-GT assays with two samples, EG09-012 (511 copies per reaction chamber) and Eden08-040 (426 copies per reaction chamber), a call rate and a call accuracy of 100% was achieved (Figure 3B). For samples EG09-012 and Eden08-040, the number of copies per reaction chamber post multiplex STA with 45 SNP-GT assays was higher than post simplex STA, since post simplex STA, a 1∶45 dilution was achieved as a result of pooling of all 45 individual PCR reactions, while, post multiplex STA, a 1∶20 dilution was performed. The data clustering was better for samples with a higher yield obtained using modified CTAB method compared to samples extracted using the Maxwell^®^ Kit but this did not affect the final genotype call once STA was performed. These results suggest that DNA samples with sub-optimal quantity can be successfully genotyped following multiplex STA.

**Figure 4:**
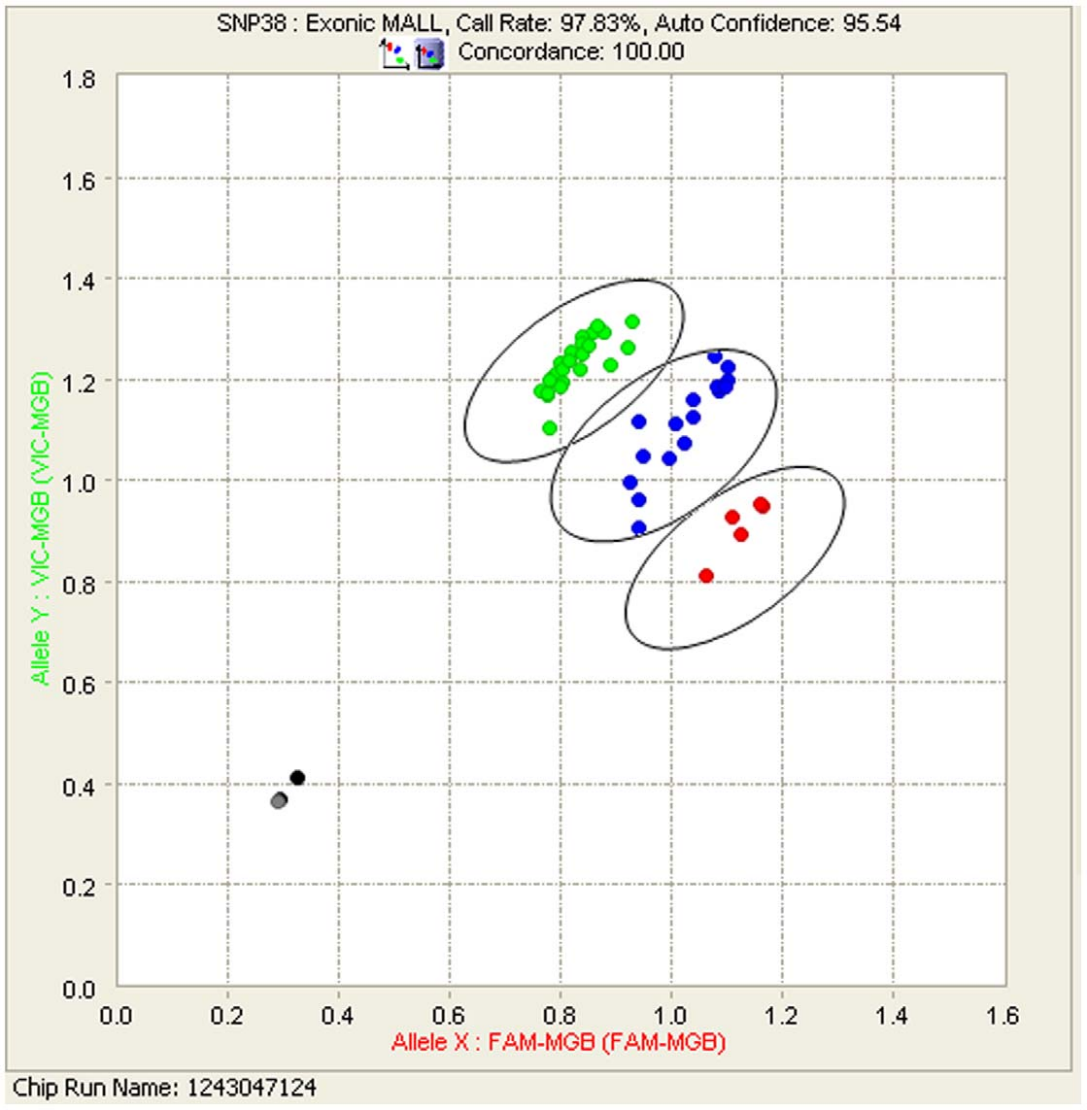
Scatter plots showing genotype call clusters for 46 whale samples using one assay (Exonic MALL). The groups (**pp**-‘**red**’, **qq**-‘**green**’ and **pq** ‘**blue**’) are denoted in circles.

In this study one additional observation was made, clustering of data points relies on specificity of the probes. The GT call data is represented as clusters on a scatter plot using the standard format of Cartesian display[17]. Cartesian coordinates use the end-point fluorescence intensities acquired for each fluorophore (FAM and VIC) on the X and Y-axis to represent the X and Y allele[17]. During the multiplex validation process using either 15 or 45 different SNP-GT assays, for one particular assay (Exonic MALL), the data point clusters for the three different genotypes (qq, pq and pp) were in very close proximity making it difficult to confidently accept the assigned genotype call (Figure 4). The fluorescent intensities corresponding to all three clusters showed positive calls for both targets. Therefore the assigned homozygous calls were manually changed to heterozygous calls since the fluorescence intensities on the X and Y-axis were similar. However, once the genotype of the sample was revealed, it was evident that the manually assigned call, pq, was actually incorrect based on the sequence data. A possible explanation is that the probes (FAM and VIC) with low specificity could result in cross-reactivity leading to an incorrect genotype call (Fluidigm, personal communication).

In conclusion, in the current study we successfully demonstrated that genotype call rate is dependant on gene copy number and then implemented the validated method to genotype 45 SNPs in the humpback whale nuclear genome using a nanofluidic IFC based genotyping system. The minimum heterozygous copies required to achieve a call rate of 100% was 38 haploid human genome copies per reaction chamber. This level of accuracy was achieved by first quantifying DNA sample using dPCR to get an accurate starting copy number concentration and then by performing gravimetric dilutions prior to genotyping. This procedure resulted in predicted number of copies dispensed in each reaction chamber. Our results demonstrate that low input copy number affects the quality of data generated, in particular when a heterozygous (pq) sample is used. For samples with low starting input DNA concentrations, incorporation of STA step prior to genotyping improved the call rate and accuracy to 100%. The proposed method validation with STA enables genotyping on the 48.48GT nanofluidic Dynamic Array^TM^ with excellent call rate and accuracy with no less than 1 ng/µL starting input DNA concentration. The genotype calls obtained for twelve whale samples using the validated method showed 100% concordance with the true genotypes determined by an independent method at the AAD. The simple work-flow employed in setting up reactions on the nanofluidic Dynamic Array^TM^, combined with STA prior to genotyping proved to be an efficient, fast and accurate way for obtaining correct genotype call with high call rate and accuracy.

## Materials and Methods

### Sample/Assay Details

For evaluating the effect of input DNA copy number on genotype call rate, three human genomic DNA samples each with a different genotype for a single SNP (rs513349) [NA17317 (pp), NA17316 (pq) and NA17313 (qq)] were purchased from Coriell Cell Repositories, Camden, New Jersey. The SNP assay (rs513349, PN: 4351376) containing two different probes (FAM-MGB and VIC-MGB) targeting a single SNP on different alleles was purchased from Applied Biosystems, Foster City, CA.

For validation of simplex and multiplex STA, total DNA was extracted from 30 mg epidermis tissue biopsies obtained from six adult humpback whales, *Megaptera novaeangliae*, using two different methods, a Maxwell^®^ tissue DNA extraction Kit (Promega) and a modified CTAB protocol[18] at the Australian Antarctic Division (AAD) (Table 3). With the Maxwell^®^ Kit, the homogenized tissue was added to the automated DNA purification cartridge and DNA was eluted in 250 µL of 1× TE (10 mM Tris, 1 mM EDTA, pH 8.0). The DNA concentration was assessed on a Nanodrop 3300 (Thermo Fisher Scientific, Australia). The 45 whale SNP-GT assays were previously validated by Polanowski *et al.* [13]. The genotype identity of the whale samples was not revealed to the scientists undertaking the SNP-GT validation at NMI.

### Digital PCR Measurement of Human Genomic DNA Samples

Digital PCR analysis was performed on the BioMark^TM^ System using the 12.765 Digital Arrays^TM^ (Fluidigm, South San Francisco). A Digital Array^TM^ consists of 12 panels, each containing 765 individual reaction chambers. Three human genomic DNA samples were retrieved from −80°C and allowed to thaw at room temperature. The tubes were then incubated at 60°C at 800 rpm for 2 min in Eppendorf thermomixer, cooled to room temperature and briefly centrifuged. An aliquot (∼40 µL) was pipetted into a polypropylene microcentrifuge tube (PN: MCT-175-C-S, Axygen INC, Union city, CA) and kept at 4°C overnight. Prior to gravimetric dilutions the samples were retrieved from 4°C and incubated at 60°C at 800 rpm for 2 min in an Eppendorf thermomixer, cooled to room temperature and analysed for UV absorbance at 260 and 280 nm. Such procedure for sample preparation was previously shown in our laboratory to achieve homogenous solution[19]. Based on the concentration determined from the A_260_ value, a two-step gravimetric dilution was prepared with 1× TE_0.1_ (10 mM Tris, 0.1 mM EDTA, pH 8.0) using a calibrated Mettler Toledo XP-205 five figure balance and in-house calibrated pipettes.

The final reaction mix for a digital panel comprised ∼1150 predicted copies of DNA, 1× TaqMan^®^ fast PCR universal mastermix No AmpErase^®^ UNG (PN: 4352042, Applied Biosystems Melbourne, Australia), 1× sample loading reagent (PN: 85000746, Fluidigm, South San Francisco) and 1× SNP-GT assay (rs513349). To reduce the uncertainty from pipetting, all PCR components, excluding DNA, were pre-mixed and the final reaction mix was prepared gravimetrically by combining the DNA with the PCR pre-mix. Ten µL reaction mix was aliquoted into each sample inlet on the digital array and approximately 4.6 µL of the reaction mix was distributed throughout the partitions within each panel using an automated IFC controller-MX (Fluidigm, South San Francisco). Each DNA preparation was analysed in triplicate or quadruplicate using duplex conditions. The No Template Control (NTC) containing 1× TE_0.1_ buffer in place of DNA was analysed in a single panel. PCR was performed using a modified fast thermocycling condition: 2 min at 95°C, followed by 45 cycles of a 2-step amplification profile of 10 s at 95°C and 30 s at 60°C.

### TaqMan^®^ SNP-genotyping Protocol

**Figure 5:**
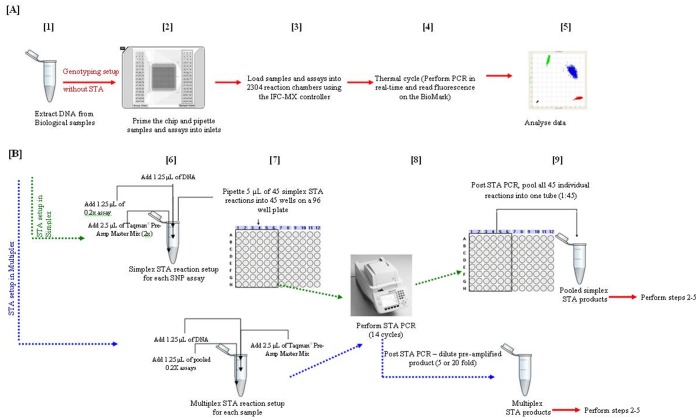
Genotyping analysis workflow with and without STA. **[A]** Steps 1–5 (denoted in red arrows) correspond to TaqMan^®^ SNP-GT protocol without STA or following simplex or multiplex STA. **[B]** Steps 1, 6–9 corresponds to STA reaction setup in simplex and multiplex conditions. Post STA, the amplified products are pooled (simplex STA), or further diluted 5 or 20 fold (multiplex STA) prior to performing TaqMan^®^ SNP-GT setup using steps 2–5.

SNP-GT reactions were setup by preparing the assays and the samples separately according to manufacturer’s instruction[17]. For each SNP-GT assay, 5 µL of a 10× assay reaction (subsequently referred to as 10× SNP-GT assay) was prepared by mixing 2.5 µL 2× Dynamic Array^TM^ assay loading reagent (PN: 85000736, Fluidigm), 1.25 µL 40× TaqMan® SNP assay (PN: 4351376, Applied Biosystems), 0.25 µL 50× ROX (PN: 12223-012, Invitrogen) and 1 µL 1× TE_0.1_. For each sample, 5 µL of sample solution (subsequently referred to as GT-sample solution) was prepared by mixing 2.1 µL gravimetrically diluted DNA solution and 2.9 µL pre-sample mix containing 2.5 µL 2× TaqMan® universal PCR mastermix with AmpErase^®^ UNG (PN: 4304437, Applied Biosystems), 0.25 µL 20× GT sample loading reagent (PN: 85000741, Fluidigm), 0.05 µL of AmpliTaq Gold^®^ DNA polymerase (PN: 4311806, Applied Biosystems) and 0.1 µL 1× TE_0.1_. For NTC, 1× TE_0.1_ was used instead of DNA. For replicate analysis, the reaction and sample volumes were scaled up as required. Four µL of a 10× SNP-GT assay and 5 µL of GT-sample solution were loaded into assigned replicate assay or sample inlets of the 48.48GT Dynamic Array^TM^, respectively (Step 2, Figure 5A).

The 48.48GT Dynamic Array^TM^ was placed on the IFC controller -MX (Fluidigm) for loading, mixing (Step 3, Figure 5A) and partitioning of each sample / assay combination at a 9∶1 ratio (Fluidigm, personal communication) into individual reaction chambers. The final volume of each of 2,304 reaction chambers is approximately 6.75 nL[17]. However, as a result of partitioning of each sample / assay combination at a 9∶1 ratio, this equates to 6.1 nL of sample and 0.7 nL of assay dispensed into individual reaction chamber. The chip was then placed on the BioMark^TM^ instrument for thermal cycling (Step 4, Figure 5A): 2 min at 50 °C, 10 min at 95°C followed by 40 cycles of a 2-step amplification profile of 15 s at 95°C and 1 min at 60°C. The data was analysed using the Fluidigm genotyping analysis software v3.0 which produces a genotype call for each sample / assay combination (Step 5, Figure 5A). In this study, default confidence threshold (65) was used to identify the call error and the spread of data points. Confidence threshold reflects the level of confidence in the display of data points for a particular SNP assay and when a call confidence is less than the threshold, the resulting call is assigned No Call[17].

### Specific Target Amplification

STA validation was performed initially in a simplex format for each assay and then under multiplex conditions. For simplex STA, each 40× TaqMan^®^ SNP-GT assay was diluted to a 0.2× concentration. For multiplex STA, all 40× TaqMan^®^ SNP-GT assays were pooled and the mix was diluted to a 0.2× concentration. Five µL simplex or multiplex STA reaction mix consisted of 2.5 µL 2× TaqMan^®^ PreAmp master mix (PN: 4391128, Applied Biosystems), 1.25 µL DNA sample and 1.25 µL of either an individual 0.2× TaqMan^®^ SNP-GT assay or the 0.2× TaqMan^®^ SNP-GT pooled assay mix (Step 6, Figure 5B). In the STA negative control (SNTC) used to monitor for false positives (Step 6, Figure 5B), 1× TE_0.1_ was used instead of DNA.

The 5 µL individual STA reaction mixes were pipetted into separate wells of a 96 well plate (Step 7, Figure 5B) and STA was performed on the Eppendorf ep ‘S’ mastercycler (Step 8, Figure 5B) with 10 min at 95°C followed by 14 cycles of a 2-step amplification profile of 15 s at 95°C and 2 min at 60°C. After simplex STA, the individually amplified products were pooled into one tube while after multiplex STA, the reaction was diluted 5- or 20- fold (Step 9, Figure 5B) prior to analysis using the TaqMan^®^ SNP-GT protocol (Steps 2–5, Figure 5A).

### Effect of DNA Copy Number on the Genotyping Call Rate for Three Human DNA Samples

To evaluate the effect of input DNA copy number on genotype call rate, sufficient GT-sample solution and 10× SNP-GT assays were prepared. Three human DNA samples from Coriell were each gravimetrically diluted based on the concentration measured by dPCR to achieve approximately 3.8×10^4^, 1.8×10^4^, 3.3×10^3^, 1.6×10^3^ and 3.1×10^2^ copies/µL (haploid genome equivalents) which equates to 116, 56, 10, 5 and 1 ng/µL. These dilutions and NTC were loaded into the Dynamic Array^TM^ in triplicate resulting in approximately 97, 46, 9, 4, 1 copies(y), respectively, per reaction chamber (estimated using equations 1–6 in Table 1). Four µL of the 10× SNP-GT assay was loaded into sixteen separate assay inlets evenly spaced across the 48.48GT array. The remaining inlets were loaded with a No Primer/Probe Control (NPC) in which the 40× SNP-GT assay (rs513349) was replaced with 1× TE_0.1_. NPC was used to monitor for cross contamination in the assay mix and also to test for possible leakage of assays between adjacent reaction chambers.

To test the effect of STA on samples with a low copy number, the DNA samples diluted to 3.8×10^4^, 1.8×10^4^, 3.3×10^3^, 1.6×10^3^ and 3.1×10^2^ copies/µL (haploid genome equivalents) were subjected to simplex STA using SNP-GT assay. After STA, samples were diluted 20- fold resulting in 2.0×10^4^, 9.5×10^3^, 1.7×10^3^, 8.6×10^2^ and 1.6×10^2^ copies per reaction chamber and analysed in triplicate using the TaqMan^®^-SNP-GT protocol (Steps 2–5, Figure 5A).

### Effect of DNA Copy Number on Genotyping Call Rate for Heterozygous Human DNA Sample

Human DNA sample NA17316 was gravimetrically diluted based on the dPCR-measured concentration to approximately 38, 18, 7, 4 and 1 copies(y) per reaction chamber. Each dilution of GT-sample solution was loaded into nine replicate inlets. Three sample inlets were treated as NTC, which contained 1× TE_0.1_ in place of DNA. Four µL of the 10× SNP-GT assay was loaded into sixteen assigned assay inlets evenly spaced across the 48.48GT array. The remaining assay inlets were loaded with NPC. The PCR thermal cycling conditions were the same as described in the TaqMan^®^ SNP-GT protocol.

### Effect of DNA Copy Number on the Genotyping Call Rate with STA for Whale DNA Samples

The process for validating the STA method in simplex and multiplex format is illustrated in Figure 5B (Steps 6–9). STA initially used fifteen SNP-GT assays previously validated by Polanowski *et* *al.* [13] using real-time PCR and was subsequently adapted for 45 SNP-GT assays. Simplex STA validation was conducted using 15 SNP-GT assays on three samples (EG09-004, EG09-012 and Eden08-040) extracted using modified CTAB protocol[18] with input DNA concentrations ranging between 8.75 and 30 ng/µL (2 fold dilution from stock, Table 3). After STA, 15 individual STA reactions for each sample were pooled and further diluted three-fold to achieve a total dilution of each individual STA reaction of 1∶45. This dilution was designed to be equivalent to the dilution that would result from pooling 45 individual STA reactions. The pooled STA reaction for each sample was serially diluted and four dilutions analysed in triplicate using the same 15 SNP-GT assays and the TaqMan^®^-SNP-GT protocol (Steps 2–5, Figure 5A). The final copies per reaction chamber were derived using equations 1–3, 4^♦^, 5–6 (Table 1). Based on equations (1–6), Table 4 illustrates an example of the copy number estimates generated without STA or with either simplex or multiplex STA using 15 SNP-GT assays. The predicted copies per reaction chamber were 17 to 662, 58 to 2270 and 49 to 1900 for EG09-004, EG09-012 and Eden08-040, respectively; the difference in the ranges being attributed to the variable starting DNA concentrations prior to STA (Table 3).

**Table 4:** Estimated DNA copy number in the reaction chamber with (simplex or multiplex) and without STA using whale genomic DNA.

	*E_RC_*		
Input DNA (ng/µL)	Simplex –STA*	Multiplex –STA*	Without STA
1	230	680	1
5	1100	3400	4
25	5700	17000	21

*The DNA copy number in the reaction chamber (E_RC_) was estimated using equations (1–6) derived in Table 1.*

* *The copies/reaction chamber post-simplex and multiplex STA PCR is an estimate obtained when using 15 SNP-GT assays with a 5 –fold dilution post STA.*

Multiplex STA validation was undertaken on all twelve whale DNA samples (6 samples extracted using Maxwell^®^ tissue DNA extraction Kit and same 6 samples extracted using modified CTAB), one NTC, one NAC, and three positive controls that had been pre-validated during the simplex STA step (EG09-012, EG09-004 and Eden08-040). After multiplex STA, each sample was diluted 20-fold to reduce the concentration of the multiplex primers prior to TaqMan^®^ SNP-GT.

For simplex and multiplex STA validation using 45 SNP-GT assays, the standard STA protocol was followed (Steps 6–9, Figure 5B) using two samples (EG09-012 and Eden08-040). Each reaction was analysed in ten replicates against 45 SNP-GT assays using the TaqMan^®^ SNP-GT protocol (Steps 2–5, Figure 5A). NTC, SNTC or NPC inlets containing 1× TE_0.1_ buffer in place of DNA and primer/probes were analysed in one or more alternate inlets. Blank inlets were used in order to accommodate the chip-setup; they contained 2.5 µL 2× TaqMan^®^ universal PCR mastermix with AmpErase^®^ UNG (PN: 4304437, Applied Biosystems) and 2.5 µL of 1× TE_0.1_ and were different to NTC or SNTC.
